# Action-projection in Japanese conversation: topic particles *wa, mo*, and *tte* for triggering categorization activities

**DOI:** 10.3389/fpsyg.2015.01113

**Published:** 2015-08-26

**Authors:** Hiroko Tanaka

**Affiliations:** Department of Sociology, University of EssexColchester, UK

**Keywords:** conversation analysis, anticipatory completion, preemptive action, projectability, Japanese conversation, topic particle *wa*, membership categorization device, set theory

## Abstract

Conversation analytic work has revealed how anticipatory completions and preemptive actions can offer invaluable glimpses into the cognitive, contextual, grammatical, and temporal bases of projectability in turn-taking, by virtue of their potential not only as a display of participants' online prediction of roughly what it might take to complete a turn-in-progress but also to plan the next move. While the predicate-final word order and the incremental transformability of turns in Japanese generally lead to delayed projectability of turn-endings, this may be partially offset by the capacity of certain postpositional particles to trigger and propel prospective action trajectories. This article engages in a case study of the topic particle *wa* (and related particles *mo* and *tte*), by demonstrating how its grammatical affordances, the categorization activities, and cognitive processing it can set in motion, coupled with the immediate contextual, and temporal-productional features may coalesce to a point of critical mass, thereby enhancing the projectability of the not-yet-produced trajectory of the current turn. The discussion attempts to contribute to recent debates on ways language-specific lexicogrammatical resources are deeply interlinked with the types of opportunities that are provided for social action.

## Introduction

### The phenomenon

The aim of this article is to demonstrate the potential for the situated use of the topic particle *wa* in Japanese conversation to serve as a powerful resource for locally projecting the possible trajectory of a turn-in-progress by activating and implementing a range of cognitive operations involving categorization activities. I focus mainly on the particle *wa* while touching upon related roles played by other particles including *tte* and *mo* (*wa* roughly glossed as “as for”; *tte* as “concerning”; whereas *mo* would be crudely equivalent to “also”). While the types of particles under consideration here are variously labeled “adverbial,” “topic” or “focus” in the literature, they will be referred to as “topic particles” for simplicity (see Section Previous Research on *wa*). The abovementioned capacity of *wa* to strongly project action trajectories may be mobilized by participants in order to trigger and propel forward anticipatory completions and even preemptive actions through engaging in categorization activities, leading to a classification or re-classification of the universe of discourse.

As an illustration, in the following excerpt, the contingent use of *wa* (line 2) provides an opportunity for a coparticipant to implement a preemptive response (line 3). Four women have been asked to discuss their preferences in men. A participant L has characterized “narcissistic men” as “fun” to have as friends.

(1) [Sakura 07] Preferences


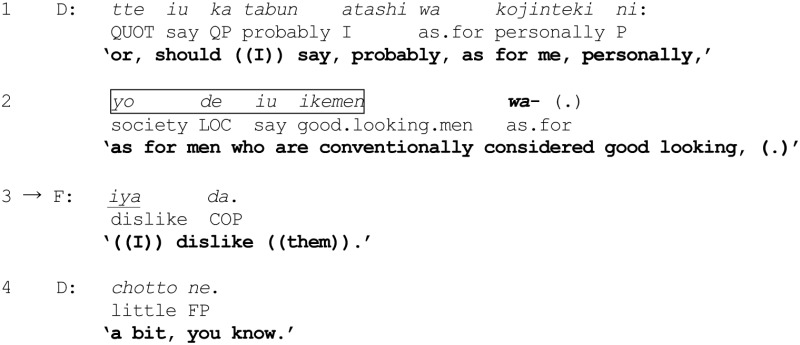


In line 1, D begins to formulate an assessment, by using *atashi* “me” and *kojinteki ni* “personally” to set the frame for the ensuing talk (Ono and Thompson, 2003, p. 332). She then introduces a referent “men who are conventionally considered good looking” (highlighted by a border) which is marked with *wa* (line 2). On hearing this turn-beginning (lines 1–2), F preemptively proffers her *own* assessment of the referent (line 3) by appropriating the grammatical slot made available by D's turn-beginning and constructing her turn as a grammatical continuation^1^. In other words, what D is projecting is being treated as so apparent that, for all practical purposes, it is seen to be sufficient not only for grasping D's intended action but for going one step further to formulate a response to it. D endorses F's action (line 4), thereby confirming F's understanding as implied in line 3. Through a close scrutiny of instances such as this where a *wa*-marked “reference formulation” (see Ford et al., 2013) triggers anticipatory completions or preemptive actions, I hope to shed light on the synergistic effect of the contextualization work performed by prior talk, the proximate temporal-productional features, and the grammatical and cognitive operations implemented by *wa*, for cumulatively laying the groundwork for augmenting the projectability of emerging turns.

The database for this study comprises approximately 20 h of telephone conversations and audio- or video-recorded face-to-face interaction among native speakers of Japanese, mainly from the Kanto or Kansai regions. Some of the data, including the Sakura corpus, are from publically available databases from TalkBank (MacWhinney, 2007), and relevant segments have been retranscribed by the author. Other data were collected by several different researchers in accordance with recommendations pertaining to human subjects of the local review boards of the universities to which they respectively belong. In each case, informed consent was freely given by all participants, and the data collected have been handled according to the Statement of Ethical Practice for the British Sociological Association (March 2002), including guidelines for the sharing of data collected for reuse in other projects. The excerpts selected for presentation in this article are drawn from the following conversations:

1. Sakura 07, Sakura 13, YKH 1, YKH 2 (video recordings of multi-party conversations)
2. IMD (telephone conversation)
3. Wedding Planning, MFriends (audio recordings of multi-party conversations)

Although space constraints limit consideration to nine excerpts, they are representative of recurrent patterns observed in the larger database. Please refer to the Supplementary Material for transcription notations and set-theoretic symbols used in this article. In the excerpts, boldface is used to highlight the topic particles under consideration, and the referents they mark are encased in a 
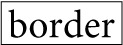
.

### Japanese conversational grammar and projectability

This article builds on work in “interactional linguistics” (e.g., Ochs et al., 1996; Selting and Couper-Kuhlen, 2001; Thompson and Couper-Kuhlen, 2005; Couper-Kuhlen and Ono, 2007) and “projectability” in Japanese. Prior research has investigated the role of various grammatical elements for action projection in Japanese: a limited list including connectives (Mori, 1999), conjunctive particles (Hayashi, 1999; Lerner and Takagi, 1999; Tanaka, 1999), adverbials (Tanaka, 2001a), adverbial and case particles (Tanaka, 1999, 2005), complementizers (Maynard, 1993; Hayashi, 1997; Matsumoto, 1998; Tanaka, 2001b), final particles (Morita, 2005, 2012), postpositions (Hayashi, 2000, 2001, 2003, 2004), predicate-final structure (Nakamura, 2009), and micro-segmentation of units (Iwasaki, 2008, 2009, 2011, 2013a).

The above works show that projectability is closely connected with the structures of syntactic and prosodic resources of the language. Work in conversation analysis and allied perspectives in Japanese have shown that even though different word orders are preferred depending on the type of social action a turn is performing (e.g., Ono and Suzuki, 1992; Tanaka, 2005), there is nevertheless a predicate-final orientation in Japanese in the sense that the production of a predicate component is normatively treated as a possible transition-relevance place (Tanaka, 1999, 2000; Nakamura, 2009). Given that the action of a turn is often embodied within the predicate (Thompson and Couper-Kuhlen, 2005), the projectability of turns in Japanese is regularly delayed until the predicate has been produced (Fox et al., 1996; Tanaka, 1999). The limited projectability of turn-trajectories, however, is to some extent offset by the pervasive use of certain postpositional particles—“case” and “adverbial” particles in particular—which serve as resources for incrementally projecting the potential unfolding of a turn-in-progress (Tanaka, 1999). Case and adverbial particles are devices that retroactively specify the grammatical sense of the immediately preceding nominal that it “marks” (e.g., as a subject, topic, object, indirect object, etc.), and “establish a grammatical linkage with that nominal to form constituents of the form [nominal + postposition]” (Hayashi, 2004, p. 348).

**Figure 1:**
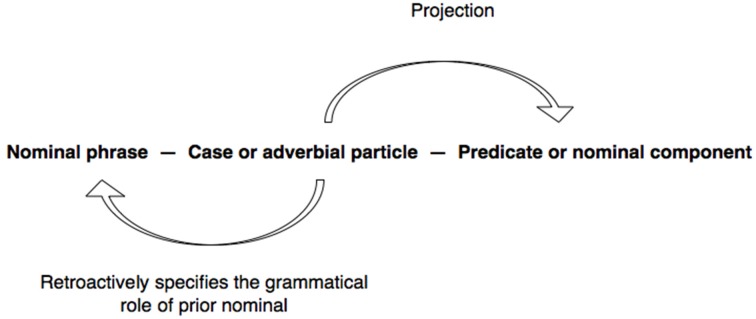
**Projective and retroactive properties of case and adverbial particles**. (Adapted from Hayashi, 2004, p. 350; Tanaka, 1999, p. 155).

Furthermore, case and adverbial particles (including topic particles) have the additional property of projecting some nominal or predicate component (a predicate in the case of topic particles) which may follow the particle within the local interactional environment (Tanaka, 1999), as schematized in Figure 1.

Applying Figure 1 to excerpt (1), D's production of the referent *yo de iu ikemen* “men who are conventionally considered good looking” (line 2) together with the attachment of *wa*, forms a reference formulation NP + *wa* “as for men who are conventionally considered good looking,” which serves a dual purpose here. First, the reference formulation provides a basis for F to predict a possible predicate that is being projected. Second, F proffers an agreement with what is predicted *Iya da.* “((I)) dislike ((them)),” which is grammatically fitted as a continuation of the reference formulation.

Even though the marking of a nominal phrase with a topic particle thus opens up a grammatical slot for a forthcoming predicate, it is not always possible to project or predict with accuracy the kind of predicate that may be supplied (Tanaka, 1999, pp. 177–182). To wit, in spite of the ubiquity of *wa* within conversational interaction, most instances of *wa* do not in fact occasion anticipatory completions or preemptive actions. Despite the key role of grammar in turn-projection, it should be underscored that it is only one out of the range of resources coparticipants mobilize for predicting the possible turn-trajectory, most significantly the immediate interactional environment, sequential context, and productional features of the turn-in-progress (Lerner, 1991, 1996, 2004; Liddicoat, 2004).

Studies on the social actions performed by postpositional particles have frequently touched upon the utility of *wa* for projecting the unfolding trajectory of turns (Hayashi, 1999, 2000, 2001, 2004; Tanaka, 1999, 2005). However, there is little in-depth research in conversation analysis specifically on the interactional role of *wa* or on the possibility that its projective capacity may hinge on the situated categorization activities it may be used to implement (but see Takagi, 2001).

### Previous research on *wa*

The potential roles and functions of *wa* in Japanese discourse have been discussed extensively by linguists, and have been at the center of countless debates, though a majority of the claims are based on invented or non-interactional data (see Shibatani, 1990, pp. 262–280; also Kuno, 1973; Clancy and Downing, 1987; Iwasaki, 1987, 2013b; Martin, 1987; Suzuki, 1995; Kaiser et al., 2001; Wlodarczyk, 2005, etc.). Shibatani (1990, p. 338) refers to *wa* and *mo* as “topic particles.” Kaiser et al. classify *wa* and *mo* as “focus particles” but distinguish the two by suggesting that whereas *mo* focuses on the nominal that it follows, *wa* primarily focuses on the predicate that follows (Kaiser et al., 2001, p. 577). They add, “*wa* is often called a topic P (particle), because it typically marks the topic of a topic-comment type S (sentence). The focus in these S again is on the comment or pred(icate).” (Kaiser et al., 2001, p. 577, parentheses added). In relation to broader grammatical groupings, Tsujimura (1996, p. 134) sees the topic particle *wa* as a type of case particle, but Shibatani (1990) distinguishes case from adverbial particles, and classifies *wa, mo*, and *tte* as adverbial particles. The particle *tte* is variously called a “quotative particle,” a “definition particle” (Kaiser et al., 2001) or a “complementizer” (Matsumoto, 1998). Depending on the particular usage, it has been described as being equivalent to other forms such as *to, to iu, to iu no*, or *to iu no wa* (see Kaiser et al., 2001).

In a well-known work, Kuno (1973, pp. 44–49) posits two types of *wa*: the “thematic” and “contrastive” *wa*. This position is contested by Shibatani, who argues that both functions can be subsumed under the rubric of the contrastive *wa*, but that the contrast “only becomes apparent when a parallel or contrasting proposition exists overtly or covertly” within the discourse environment (Shibatani, 1990, p. 265). Others like Martin (1987, pp. 60–65) and Kaiser et al. (2001) enumerate multiple usages for the particle, while noting that one such usage is to mark contrasts. For instance, according to Kaiser et al. (2001, p. 582), when a comment is made on a nominal (phrase) marked with *wa*, it “implies that the comment may not apply to other” nominal (phrases). This raises the issue of specifying the kinds of “other nominals” that the comment would be inapplicable to. Another frequently reported feature of *wa* is that its usage and that of the particle *mo* are “mutually exclusive” (Takeuchi, 1999, p. 133). While *wa* is purported to have the general characteristic of “excluding” the nominal phrase that it marks, *mo* is described as “inclusive” and is translated as “too” or “also” (Kaiser et al., 2001, p. 242).

Maruyama (2003) addresses some of the issues indicated above by examining the function of *wa* in naturally occurring conversation, focusing on the importance of the discourse context in which *wa* occurs. She reports that a majority of cases of *wa* in her data fall into two main types of schemata, both of which mark a contrast (an opposite or parallel relationship) in some way: in the first type, given a component Y which contains a *wa*-marked nominal, attention on the discourse context prior to Y yields components X which stand in a semantically contrastive relation to Y; as for the second type, likewise given a component Y containing a *wa*-marked nominal, the discourse context prior to Y will contain a Set X comprising various components from which the component Y is specifically being singled out. With respect to the latter type, she notes that although X and Y do not stand in semantically contrastive relation, “*wa* in this schema still marks a contrast, for when Y is chosen out of the Set X by a speaker, Set X and Y are in a contrastive relationship in the sense that only Y is chosen” (Maruyama, 2003, p. 268). It is becoming common in recent commentaries on *wa* to incorporate the concept of “sets” within the explanatory apparatus, as exemplified by Shoichi Iwasaki's characterization of the contrastive function of *wa* to mark a referent to “represent an entity that is set off against another entity of the same class…due to their different attributes, which nonetheless constitute a coherent set” (Iwasaki, 2013b, p. 244).

Research in interactional linguistics is increasingly converging on the notion that postpositional particles primarily have a pragmatic rather than a grammatical role (e.g., Ono et al., 2000 on *ga*). Following in this vein, Takagi's (2001) study of child-adult interaction focuses on the use of *wa* in question formulations of the form “referent + *wa*?” (which she refers to as “*wa*-ending turns”). Takagi argues that a *wa*-ending turn is simultaneously deeply embedded in the particulars of the ongoing activity while at the same time prospectively oriented by inviting a recipient to supply a predicate that will be associated with the referent marked by *wa* (Takagi, 2001, p. 187). What is more, she contends that a *wa*-ending turn invariably has a directionality (not observed with other particles such as the nominative *ga* or accusative *o*) which propels the sequence forward by providing a grammatical slot for recipients to offer “what can be said about the reference,” and going beyond simply “projecting” what should come next (Takagi, 2001, p. 187). Drawing on this and other previous studies, the present article pays particular attention to the contingent treatment of *wa* as mutually displayed by participants within the dynamic moment-by-moment unfolding of talk. In doing so, insights may be gained into its extensive utility for (membership) categorization activities. It will be shown that *wa* and other topic particles are critical resources for the performance of rudimentary categorization operations.

### Membership categorization and set theory

Membership categorization (Sacks, 1972, 1986) is concerned with practices used by participants in interaction to categorize people and the activities they engage in. In the process, participants display their cultural knowledge and commonsense reasoning in understanding and classifying the social world around them. Sacks points out that there are various membership categories that are used in our everyday interaction—such as the set of members of a population who are professionals. Moreover, there are certain ways in which we associate particular categories with others because they “go together” in some way—e.g., the larger class consisting of two categories, professionals and laypersons, which we associate together because they classify persons according to whether they have special rights to deal with certain types of troubles or not. Sacks calls such overarching classes “membership categorization devices” or MCDs:

By this term I shall intend: any collection of membership categories, containing at least a category, which may be applied to some population containing at least a member, so as to provide, by the use of some rules of application, for the pairing of at least a population member and a categorization device member. A device is then a collection plus rules of application (Sacks, 1986, p. 332).

An often cited example of a MCD is one defined along the dimension of “stages of life.” If we denote membership categories by using curly brackets { } and a membership categorization device through square brackets [ ], the MCD “stages of life” consisting of different membership categories may be represented by [{babies}, {toddlers}, {children}, {adolescents}, {young adults}, {the middle-aged}, {the elderly}] or through a relative measure in relation to the ego as in [{younger persons}{older persons}]. It should be noted that these collections are not analytical categories, but are invoked by participants to reflect members' knowledge as contingently formulated and locally negotiated in interaction.

As will be discussed herein, *wa* (as well as other topic particles including *tte* and *mo*) are implicated in the performance of the most primordial of membership categorization or set-theoretic operations (see Wlodarczyk, 2005). The data reveal that topic particles are employed to classify all manner of things in the physical and conceptual universe. Indeed, it has been suggested that these resources are used “indiscriminately” whether they apply to person, object or conceptual categories.

While people certainly differ from objects as stimuli, the categorization rules and conceptual structures used in person and object perception may not be fundamentally different. Moreover, to the degree that differences do exist we can, presumably, gain finer insight into person categorization systems by comparing and contrasting them against this baseline of object categorization (Cantor and Mischel, 1979, p. 8).

In order to make full use of prior research on membership categorization in conversation analysis while simultaneously drawing on notions from rudimentary set theory (e.g., Halmos, 1960), only excerpts bearing on person references and categories will be used as examples in this article, though it can be empirically established that much of membership categorization is extendable and adaptable to other types of categories and collections of categories. Thus, the term “category” will be used interchangeably with “set,” and “membership categorization device” as equivalent to the notion of the larger collection that contains the categories/sets which are associated together along some dimension.

In the half century following the inception of conversation analysis, the insights provided in Sacks' (1972, 1986) seminal work on membership categorization have been further developed by conversation analysts and ethnomethodologists (Hester and Eglin, 1997; Egbert, 2004; Schegloff, 2007a,b; Deppermann, 2011; Lerner et al., 2012; to name but just a few). The reader is referred to Day (2013) for a useful summary. The journal special issue [*Discourse Studies* 2012 Issue 14(3)] is a reflection of a renewed recent interest in membership categories.

The following sections proceed step-by-step to construct a picture of the ways in which members use *wa* (and other topic particles) for performing categorization or set theoretic operations and projecting the upcoming trajectory of talk. A range of interactional environments in which the situated marking of a referent with *wa* triggers anticipatory completions or preemptive actions will be examined, suggesting a close interconnection between the kinds of categorization work that *wa* can perform, the nature of the prior contextualization work, and the temporal-productional features of talk.

## Basic categorization/set-theoretic actions performed by topic particles

Before narrowing the focus to the role of *wa*, it would be useful to gain a sense for how members may deploy a range of topic particles as interactional resources depending on the kind of categorization activity to be implemented. I begin with *tte* as a typical example of a topic particle that can contribute to laying the groundwork for further categorization activities, and go on to discuss the mutually exclusive uses of *mo* and *wa*. The particle *tte* shares with other topic particles the general characteristic of marking a referent and projecting a predicate. It will be shown that one of the relevant activities *tte* may engender is to topicalize the incumbency of a referent in some category.

The way *tte* operates on the parameters “referent” and “category” is illustrated in the following excerpt taken from a telephone conversation between fellow alumni from high school, Ken and Mai. Ken has called Mai to tell her about a grandiose wedding reception he attended recently in which Yoko, a common friend of the two from high school, was the bride. From an earlier part of the conversation, it is clear that Yoko is a medical doctor, and that she is marrying another doctor from the same university hospital. Immediately before the part shown, Ken has been describing the guests attending the reception. There is something in Ken's telling which Mai notices as departing from her presupposition, as indicated by her turn-initial *eh!* in line 1 (see Hayashi, 2009).

(2) [IMD 254] Doctors


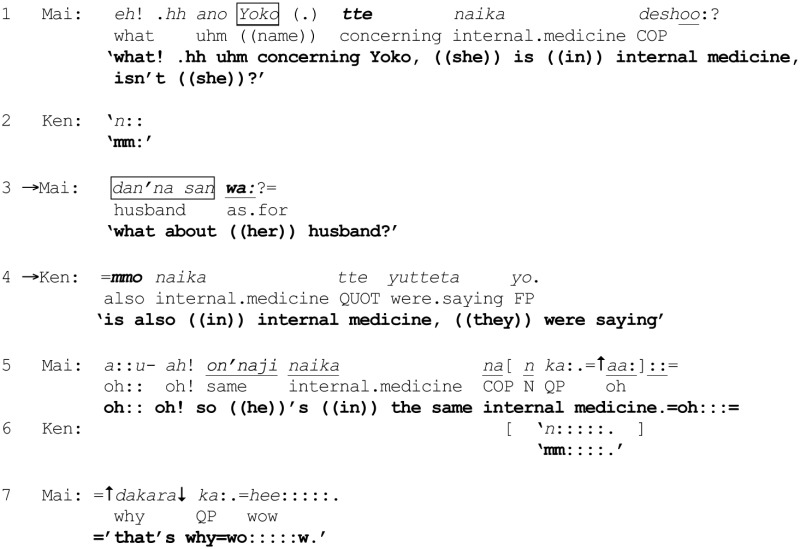


Mai's question in line 1 is tantamount to asking for confirmation that Yoko is an incumbent of the category {doctors of internal medicine}. The capacity of *tte* to invoke the relevance of membership in a category draws in part on “the economy rule” that “if a member uses a single category from any membership categorization device, then they can be recognized to be doing *adequate reference* to a person” (Sacks, 1986, p. 333).

The use of *tte* for assigning membership of a referent in a category concomitantly proposes “classifying things” as a relevant activity to be engaging in within the local context, as demonstrated by the regularity with which such instances either engender, or are used as a preliminary to, some main categorization activity. Once the groundwork is established, co-participants can exploit it as a framework to engage in further categorization activities, by activating “the consistency rule”: “If some population of persons is being categorized, and if a category from some device's collection has been used to categorize a first member of the population, then…other categories of the same collection *may* be used to categorize further members of the population” (Sacks, 1986, p. 333). In the present case, line 1 sets the stage for classifying another member, as instantiated by Mai's main query (line 3) to be examined closely below. Although it is not possible to elaborate here, other topic particles such as *toka* (see excerpt (7) line 4) and even *wa* (see excerpt (8), line 6) may likewise be used for proposing categorization as an activity to be pursued.

Crucially, this excerpt also illustrates the mutual incompatibility of the operations performed by *wa* and *mo* respectively, at least in the specific context where a category (in this case, {doctors of internal medicine}) has just been invoked. Specifically, after receiving the sought-after confirmation that Yoko specializes in internal medicine (line 2), Mai next proceeds to ask about Yoko's husband, *dan'na san* wa: “what about ((her)) husband?” (line 3), by using a question formulation that exploits the projective properties of *wa*. As noted in Section Previous Research on *wa*, this use of *wa* serves as “an invitation to provide what can be said about the reference in the *wa*-ending turn” (Takagi, 2001, p. 187). First, lines 1 and 3 taken together propose that Mai knows that the husband is likewise a medical doctor but not his specialty, since it is the specialty that is the target of the query. Furthermore, it can be argued that the employment of the *wa*-ending turn, *dan'na san* wa: in this specific sequential context, namely, immediately following the invocation of a category, exhibits Mai's presupposition that the husband is more likely than not to have a different specialty from that of Yoko—i.e., that the husband is potentially a member of a category {doctors of specialty *Y*} where *Y* is unspecified but different from internal medicine. The possible tilting toward the husband belonging to a different category than the one already invoked (i.e., internal medicine) is partly indicated by the fact that Mai does not use the equally accessible alternative question formulation *dan'na san mo?* “The husband also?” [see Excerpt (3) line 5 for an example], as well as by the way Mai subsequently responds to Ken's answer to the question. Further evidence of the potential tilting toward a different category of this situated use of *wa* will be examined below.

But first, we see that Ken goes on to respond that the husband is *also* in internal medicine (line 4), by countering Mai's presupposition. In order to do this, he has been forced to adopt a turn-beginning that avoids the particle *wa*, which can be used to project possible “exclusion” of the husband from the category {doctors of internal medicine} (line 3), and instead, to use *mo* which projects “inclusion” in the same category (line 4). By beginning with *mo*, Ken constructs a “postposition-initiated utterance” (Hayashi, 2000, p. 215ff) which connects with the same referent *dan'na san* in Mai's query (line 3) and now marks it with *mo* (line 4), thereby altering the trajectory of the turn:


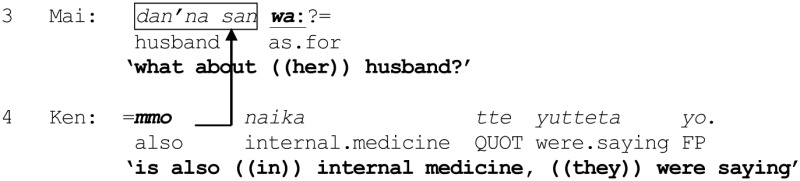


The procedure results in marking *dan'na san* not with *wa* but *mo*:


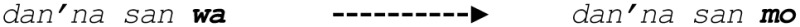


now enabling Ken to project with “consistency” that the husband's specialty is the same as Yoko's. That Mai may have not even contemplated such a “coincidence” (when she initiated her enquiry through the use of *wa* in line 3) is displayed in her uptake in lines 5 and 7: through the repeated deployment of *aa* “oh” to index a “change of state” (Heritage, 1984) from not knowing to being informed; through commentary attributing the “change of state” to the revelation that the husband is likewise in internal medicine; and finally, through the interjection *hee* “wow,” proposing that Ken's informing has resolved some incongruity that had been puzzling her in line 1 (see Tanaka, 2013). These observations reinforce the possibility that a question formulation *x wa?* immediately following an invocation of a category *Y* may contingently be tilted toward an answer that excludes *x* from the category *Y*, although further work is needed to explore its workings in other local contexts.

**Figure 2:**
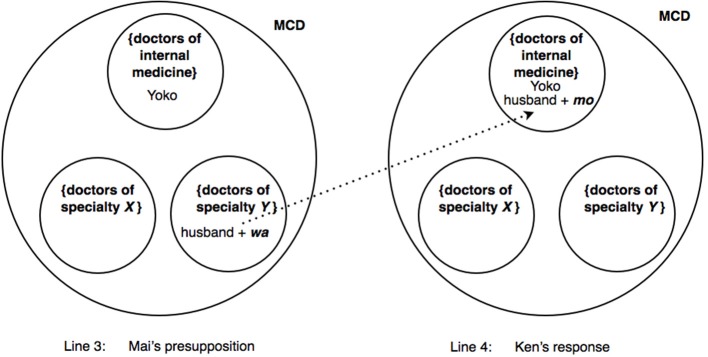
**Excerpt (2): In enquiring about the husband's specialty, Mai marks “the husband” with *wa*, which has a tilting toward exclusion from {doctors of internal medicine} and toward inclusion in {doctors of specialty *Y*}, where *Y* is unspecified (left)**. Ken responds by marking “the husband” with *mo* to place him back in {doctors of internal medicine} **(right)**. MCD = “types of medical doctors” = [{doctors of internal medicine}, {doctors of specialty *Y*}, {doctors of specialty *X*}, …], where *Y* and *X* are unspecified. Only three of the many possible specialties have been represented in the figure.

Thus, in terms of categorization activities, lines 3 and 4 exemplify three basic operations performed by *wa* and *mo*. First, by deploying *wa* to mark the referent *dan'na san* “husband,” Mai potentially excludes the referent from the already invoked category {doctors of internal medicine} *and* suggests that the husband may belong to a different, though unspecified category {doctors of specialty *Y*} which Ken is invited to name. Second, Mai's deployment of *wa* additionally invokes an overarching membership categorization device “types of medical doctors” in which the respective categories to which Yoko and her husband may belong to are co-class members, through an application of the “consistency rule.” Third, whereas Mai's turn (line 3) potentially places the husband outside the category {doctors of internal medicine}, Ken returns the husband in the category {doctors of internal medicine}. The entire process is schematized in Figure 2.

As a further demonstration of the differential usages of *wa* and *mo* and the possible tilting of a *wa*-ending question formulation toward a category different from one that has been invoked, I reanalyze Excerpt (3) from Takagi (2001), which shows a very young child Y (2 years and 4 months) switching between *wa* and *mo* to index her evolving expectations as to who (among the people present in the room) may be participating in a planned visit to her grandparents' house in a few days time—i.e., inclusion or exclusion from the category {people who are going on the visit}. The little girl is asking her mother M as to who will be going on the outing. *Jun-kun* is her brother (5 years and 2 months). In line 10, the child is referring to the researcher (a stranger) who is visiting for the purpose of making recordings of the family interaction. Note that –*kun* and –*chan* are informal name suffixes commonly used when addressing or referring to someone (or oneself in the case of a small child).

(3) Yacchan (from Takagi, 2001, pp. 158–159; modified translation)


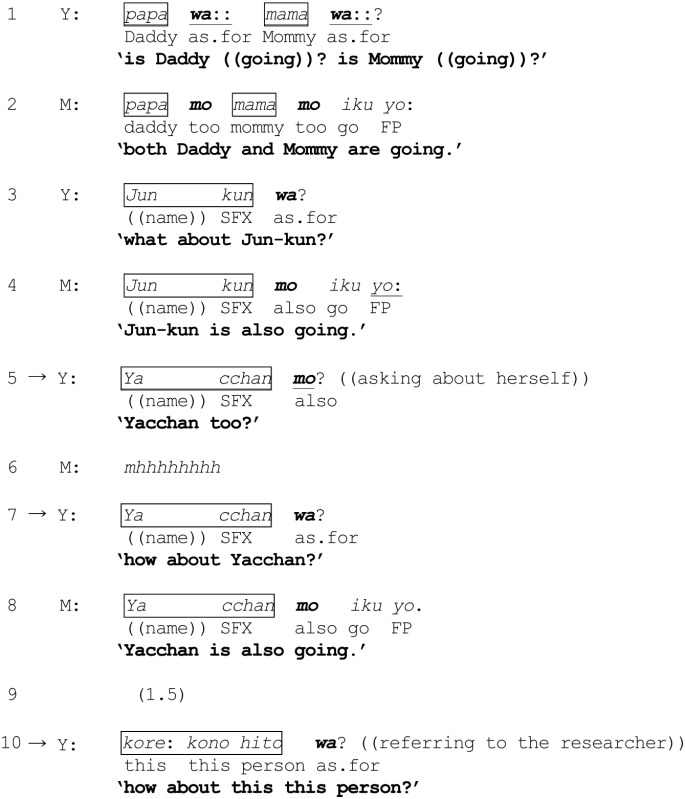


Using *wa*-ending question formulations, Y begins by asking whether her father, mother and brother are going on the visit. Once it is established that all other members of her immediate family will participate (i.e., members of the category {those going on the visit}), Y then switches to the use of *mo* (line 5) to enquire about herself, displaying a “reasonable” assumption of the likelihood of herself being included in the said category. In the absence of an immediate affirmation (line 6) however, Y “repairs” her *mo*-ending question formulation, with its tilting toward “inclusion,” to the *wa*-ending (line 7), which divests the question of such an assumption and is tilted instead toward the co-class category {those who are not going on the visit}. It nevertheless emerges (line 8) that Y was justified after all in assuming inclusion in the former category (line 8). Interestingly, Y avoids using *mo* when next enquiring about the researcher (line 10), thereby exhibiting her assumption that the researcher is unlikely to participate in the family visit. The child appears to be using *mo* and *wa* to display her differential predictions (and deductive processes) with regard to probable inclusion or exclusion: *mo* to index an expectation for a referent to be included in the previously invoked category, and *wa* for the converse (i.e., inclusion in the co-class category).

Another excerpt is considered to provide a recipient's perspective on the possibility that a question formulation *x wa?* (immediately following the invocation of some category or categories) may contain an implicit tilting toward the category incumbency of *x* in a different co-class category of the ones already invoked. In excerpt (4), a participant makes explicit his interpretation of the categorization implications of a question formulation *x wa?* Four male university students have been asked to talk about their preferences in women. The discussion has digressed from desirable character traits to physical attributes:

(4) [Sakura 13] Kindness


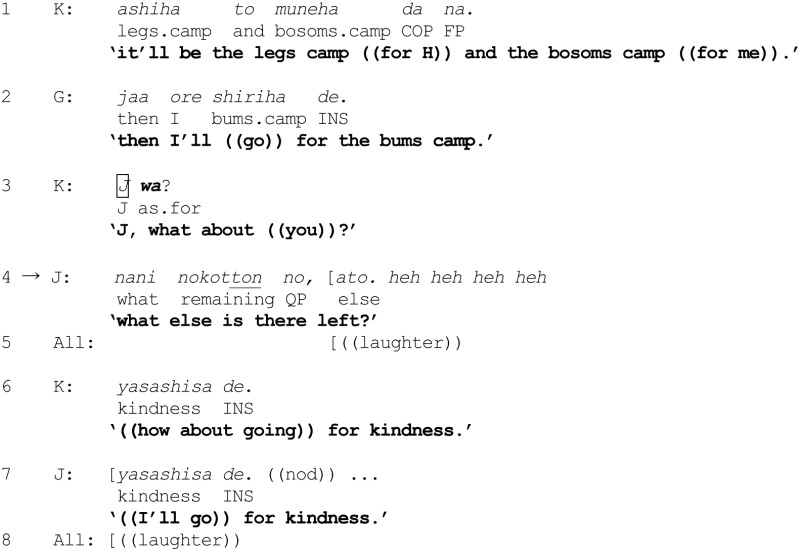


K's turn in line 1, in effect, assigns H and himself respectively to the categories “legs camp” and “bosoms camp,” in the MCD of men categorized according to their (anatomical) preferences. G follows suit in line 2, putting himself in the “bums camp,” using the connective *jaa* “then” to indicate that he has limited his choice to a not-yet-selected camp. K then turns to J through the question formulation *J wa?* (line 3). Interestingly, J responds with a playful counter-question “what else is there left?” (line 4), thereby exhibiting an interpretation of the question formulation *J wa?* as embodying an implicit expectation to select a camp (category) not claimed by the others—namely, a co-class category in the same MCD.

In (5), *wa* is mobilized in a similar interactional environment, but in this case, for implementing and confirming an understanding check. Importantly, the excerpt exemplifies the interlocutors concurring (through co-constructions) on the action potentially being projected by *wa* in the immediate aftermath of the invocation of a category, thereby making evident an implicit tilting of the employment of *wa* toward exclusion of the referent it marks from an already invoked category. W and her fiancée H are arranging the logistics of their wedding reception, guided by S, their wedding planner. S has told W and H that it is more customary to provide a single take-home gift for guests who are a married couple rather than separate gifts. To this, W has just mentioned that she knows of cases whereby wives receive alternative gifts. In line 1, she is asking about such gifts.

(5) Wedding planning


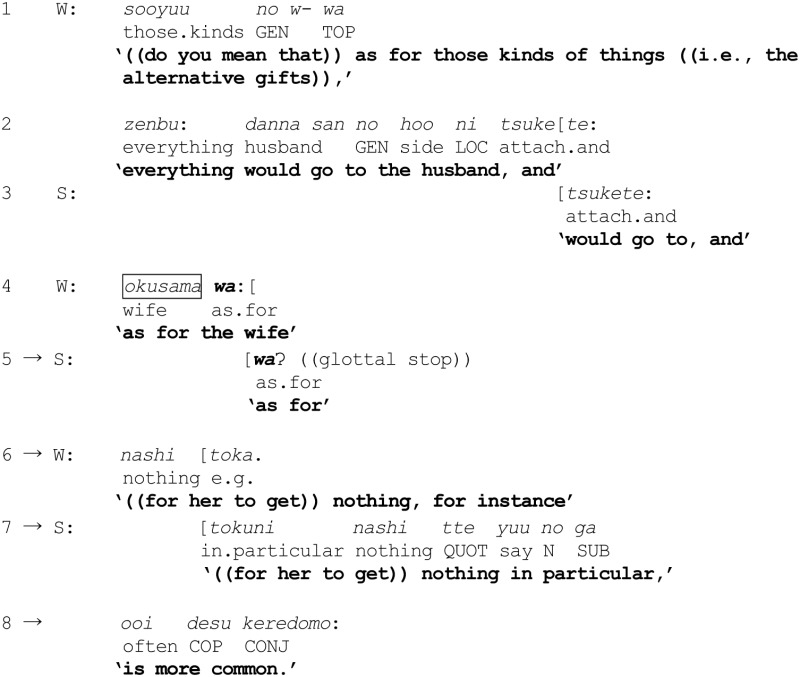


In lines 1–2, W first checks if S is implying that such alternative gifts should likewise all go to the husband—i.e., that the husband belongs to the category {guests receiving all the take-home gifts}. S affirms this through a co-construction (line 3). Then, in lines 4 and 6, deploying the reference formulation *okusama wa:*, W embarks on a further understanding check as to whether the wife tends to get nothing—namely, that the wife may belong to the co-class category {guests receiving no take-home gifts}. In lines 5 and 7–8, S affirms W's understanding again by co-constructing W's turn. A closer inspection of the intricate, moment-by-moment coordination of action here affords a rare opportunity to witness the action-projection-capacity of *wa* being ratified and jointly mobilized for implementing the categorization activity of exclusion from an already invoked category. First, on hearing W's talk *okusama wa:* (line 4), S quickly echoes simply the *wa* (line 5), thereby endorsing and herself re-mobilizing its capacity for projecting the trajectory of the ongoing turn. W, for her part, treats S's echoing of *wa* as a go-ahead to render explicit what *wa* is being used to project (line 6), duly ratified by S (lines 7–8). In other words, the speakers are collaboratively displaying and implementing their shared understanding of the use of *wa* for excluding a referent from the category which was invoked immediately beforehand.

This section has demonstrated members' orientations to *tte* as a resource to invoke a category, and the mutual exclusivity of *wa* and *mo* depending on the type of categorization activity being proposed. In brief, *wa* is contingently used to exclude a referent from a previously invoked category (which thereby makes relevant a different category in a MCD), and to assign it to a co-class category within that MCD. On the other hand, *mo* is used to mark a referent and to include it in a category which has already been invoked.

## *Wa* for triggering anticipatory completions and preemptive actions

Observations were made above concerning the types of categorization activities that may be performed through a number of topic particles. Among other things, it was shown that the use of *tte* for explicit invocation of a category and a MCD is one way of providing a foundation, which participants may build on to perform further categorization work such as exclusion or inclusion of other members of the population from the said category. Needless to say, employing *tte* is not the sole way to realize such prior contextualizing work. The instances to be considered show that critical groundwork may be laid in a variety of other ways through participants' coordinated mobilization of resources that emerge contingently within the unfolding of talk. The aim here is to explore how such preliminary activities can give rise to an interactional environment ripe for the situated deployment of *wa* that activates anticipatory completions and preemptive actions.

In contrast to the verbally explicit invocation of a category, for instance in excerpt (2) above, excerpt (6) below exemplifies how precursory categorization work may be initiated through visual conduct even when no category is named, and spark off further categorization activities. Moreover, it provides additional empirical support for the mutually exclusive operations of *wa* and *mo*. Recall that in excerpt (2), *mo* was used in order to include a referent in a category that had already been invoked, to reverse an apparent presupposition about category non-incumbency suggested by *wa*. The converse is demonstrated here: namely, how *mo* may be replaced by *wa* in order to repair a presumption about category incumbency displayed through *mo*.

Chie has been engaging in a telling about a recent holiday at a Hawaiian theme park, which Mari has no knowledge of. Shortly before the extract, Chie has begun to describe the Hawaiian shows that were featured. In the part shown, Mari is prompting Chie to elaborate. Included in the transcript are descriptions of some visual conduct critical for understanding the categorization activities the participants implement.

(6) [YKH2, 3'43-4'02”] Hawaiian show


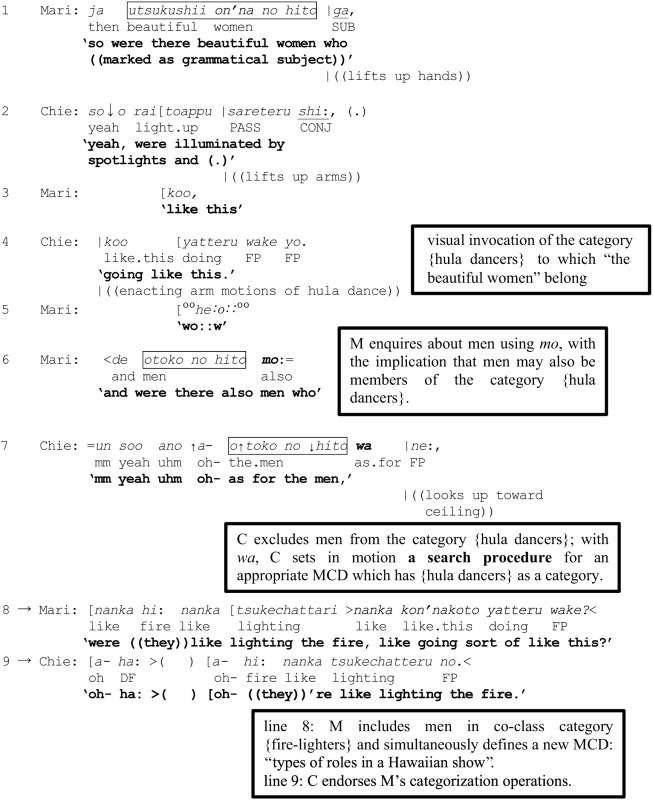


In line 1 Mari encourages elaboration by enquiring about the beautiful women, with a turn-beginning of the form “referent (the beautiful women) + particle *ga.*” As Ono et al. (2000) have shown, *ga* is regularly used to foreshadow a forthcoming description of the state of a referent. This prompts Chie to provide a description: “yeah, were illuminated by spotlights and (.) going like this.” (lines 2 and 4), portraying their state by enacting the arm motions of hula dance. In other words, through mobilization of grammar and visual conduct, Mari and Chie are characterizing “the beautiful women” as {hula dancers}.

Mari next proceeds to enquire about the men, marking a new reference formulation *otoko no hito mo* “the men” with the particle *mo*, which can potentially be heard as enquiring if the men were also doing hula dance—tantamount to including the men in the just invoked category {hula dancers}. That Chie finds the use of *mo* problematic here is revealed by what happens next (line 7). After embarking on what sounds like an agreement, Chie stops mid-turn and produces a “change-of-state” token, (Heritage, 1984), ↑*a*- “oh-,” which may be used to initiate repair (Schegloff, 1992, p. 1305). She then continues by replacing Mari's use of *mo* with *wa*:


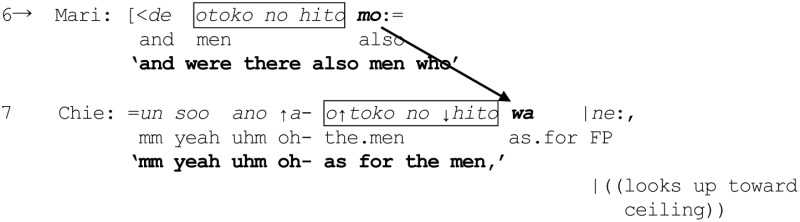


How this replacement is treated by Mari can be observed in the overlap that ensues (lines 8 and 9). First, on the basis of Chie's now revised marking of “the men” with *wa*, and without the benefit of hearing how Chie's turn develops, Mari embarks on an anticipatory completion to request confirmation that the men may instead have had a different role such as that of lighting a fire (line 8), thereby registering that she had mistakenly assumed the men were also hula dancers:


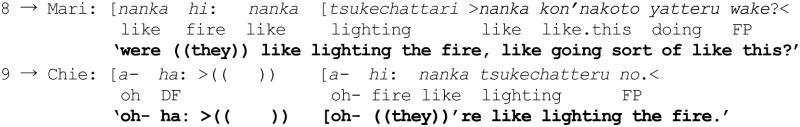


In other words, Mari's uptake in line 8 displays an understanding that Chie's marking of “the men” with *wa* is projecting a turn trajectory that excludes “the men” from the category {hula dancers}. But the *wa*-marked reformulation (line 7) indicates that Chie is simultaneously projecting something more; that Mari goes on to propose that the men may be lighting the fire attests to the fact that *wa* has apparently set in motion a “search” procedure' for a possible category—containing “the men”—which is a co-class category of {hula dancers} in some overarching MCD. Indeed, the anticipatory completion (line 8) evidences that the search has yielded the category {fire-lighters} which is a co-class category of the category {hula dancers} within a larger MCD. Although not made explicit, Mari's mention of fire-lighters evokes an MCD such as “types of roles in a Hawaiian show,” which would contain {fire-lighters} as a co-class category of {hula dancers}. Such an understanding on the part of Mari is ratified by Chie through the latter's acceptance and partial repetition of Mari's suggestion in overlap (line 9).

**Figure 3:**
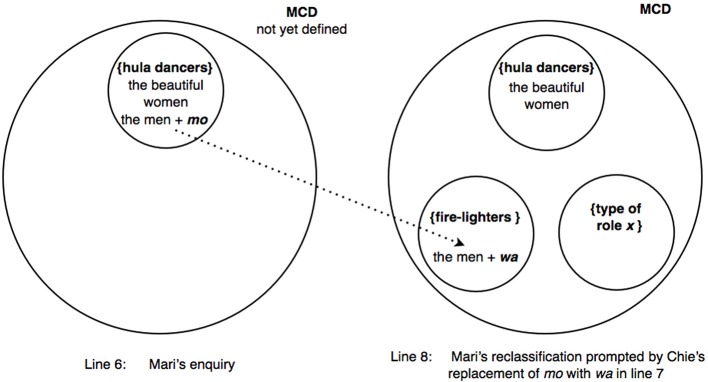
**Excerpt (6): Mari marks “the men” with *mo* to enquire if they belong to {hula dancers} (left); subsequently, Chie's marking of “the men” with *wa* prompts Mari to exclude them from {hula dancers} and to reassign them instead to {fire-lighters} (right)**. MCD on right = “types of roles in a Hawaiian show” = [{hula dancers}, {fire-lighters}, {type of role *X*},…], where *X* is unspecified. Only three of the possible roles have been represented in the figure.

An important factor enabling Mari's anticipatory completion in line 8 is arguably whether the amount of contextual information accumulated up to that point has reached a certain threshold level, thereby providing a reasonable basis for projection. In retrospect, the participants' collaborative work in invoking the category {hula dancers} and including “the beautiful women” in that category (lines 1–4) is analyzable as constituting vital preliminary steps for eventually evoking the larger MCD— “types of roles in a Hawaiian show.” Of course, whether an occasion arises for such immanent MCDs to be actively invoked is contingent on how the interaction unfolds. Here, precisely such an occasion is presented through Mari's further categorization activity to attempt to classify “the men” (line 6), taken even further by Chie's projected reclassification (line 7), synergistically thrusting the immanent MCD into the scope of interactional relevance. Figure 3 represents the reclassification resulting from Chie's replacement of *mo* with *wa* in line 7.

A final factor contributing to the anticipatory completion are productional features of Chie's turn-beginning in line 7. Chie displays an attempt to search for a description of {the men} at the end of line 7 partly through her upward glance toward the ceiling suggestive of a word-search, as well as the sound-stretch on the final particle *ne* which can be heard as a move to gain time. Such disruptions in progressivity provide “unprojected opportunities” for Mari to implement an anticipatory completion (see Lerner, 1996), but may have simultaneously given Mari just enough time to execute the cognitive operations made relevant by Chie's production of *wa*.

Incidentally, the fact that Chie targeted the particle *mo* (projecting “sameness”) in line 6 for replacement with the particle *wa* (projecting a “contrast”) in order to repair Mari's original suggestion that the men may also be engaging in hula dance, bears witness to Chie's understanding of the “inappropriateness” of using *mo* when talking about a referent *otoko no hito* “the men” supposedly not belonging to a previously invoked category {hula dancers}. To rearticulate, *wa* was used not only to exclude “the men” from the category {hula dancers} but also to enable the inclusion of “the men” in another category, which is a co-class category of the overarching MCD “types of roles in a Hawaiian show,” within the complement of the category {hula dancers}. This instance contributes toward further buttressing the potentially mutually exclusive nature of the two particles *mo* and *wa* (see Takeuchi, 1999, p. 133), and the capacity of *wa* to mobilize a search procedure for an appropriate MCD.

Consider another instance, this time of a preemptive response, which sheds further light on the operations set in motion by *wa*, the significance of prior contextualizing work, and productional features, as well the ways in which they work in tandem to permit coparticipants to form a basis for projecting the likely trajectory of a *wa*-marked turn-beginning and to respond to it. Furthermore, this instance will be used to demonstrate that the process of anticipating the kind of MCD being invoked may be vastly simplified when the *wa*-marked reference formulation projects an opposite co-class category—i.e., narrowing down the choice to just one candidate co-class category.

In this conversation [same as the one from which excerpt (1) was taken], a group of female students at university were asked to talk freely about their preferences regarding men. The participants have been discussing their likes and dislikes, exemplifying their opinions by referring to members of a popular Japanese, all-male band, including Masa and Shun, who are also topicalized in the excerpt itself. Shortly before the stretch of conversation shown below, the talk had revolved around types of eyebrows and the thicknesses of hair in men, with F expressing a dislike for certain types of eyebrows in men. E then commented that she was disinterested in the types of eyebrows men have, to which L agreed. D nevertheless went on to express her dislike for thick eyebrows, with which F agreed by citing Masa as an example. D took this further by asserting her aversion to men with thick hair. Then, in line 1, L playfully objects to everyone using the band members as exemplars of the traits.

(7) [Sakura 07, 262, Thick or thin features]


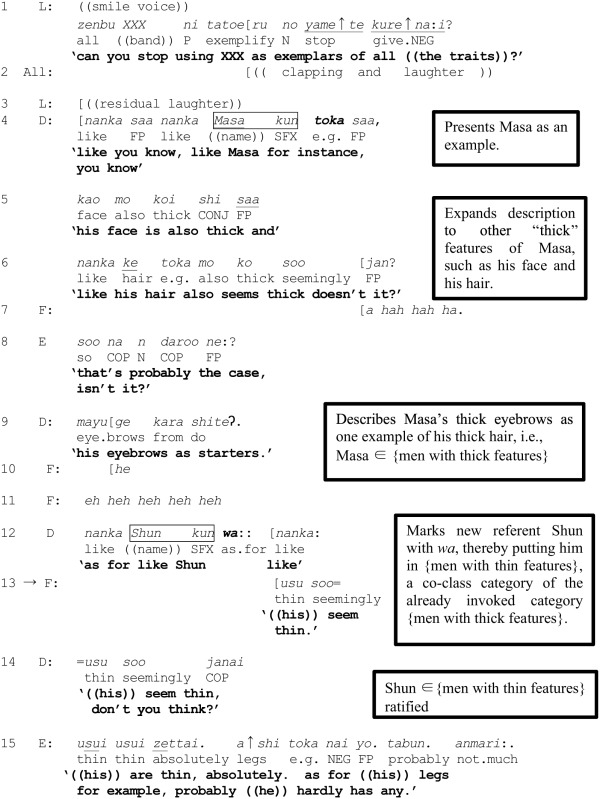


L's tongue-in-cheek plea to the others (line 1) makes explicit her judgment that the members of the band are being used as exemplars embodying the various attributes of the target population “eligible men.” L's objection notwithstanding, D proceeds (line 4) to illustrate her earlier mentioned aversion by citing Masa as embodying thick features—thick (prominent) face (line 5), thick hair (line 6), and thick eyebrows (line 9). By enumerating a range of features of Masa which epitomize the quality of “thickness,” D in effect, invokes and makes relevant the category {men with thick features} to which Masa is being assigned. Note that the referent *Masa kun* (line 4) is marked with a topic particle *toka* used here to link Masa to the emerging category as one out of an unspecified number of incumbents (a usage similar to that of *tte* as detailed in Section Basic Categorization/Set-theoretic Actions Performed by Topic Particles).

Having laid the groundwork for further categorization work by making relevant the category {men with thick features} containing Masa, D then names another band member, Shun, through a *wa*-marked reference formulation (line 12). As soon as this turn-beginning *nanka Shun kun wa*:: “as for like Shun” (line 12) can be heard, F enters with a preemptive response *usu soo* “((his)) seem thin” (line 13), which is built on the prediction that D is projecting exclusion of Shun from the category {men with thick features}. F's response is ratified by D herself through repetition (line 14), and followed by an upgraded agreement and further elaboration by E in line 15. Lines 13–15 exhibit three participants' shared understandings that *Shun kun wa* locally projects a characterization of Shun as having features which are “thin” in some sense, i.e., that Shun belongs to the category {men with thin features}. This instance further substantiates the role of *wa* to assign a referent it marks to a co-class category {men with thin features} of an already invoked category {men with thick features} within an overarching MCD which partitions the universe of discourse (i.e., “eligible men” in this example).

**Figure 4:**
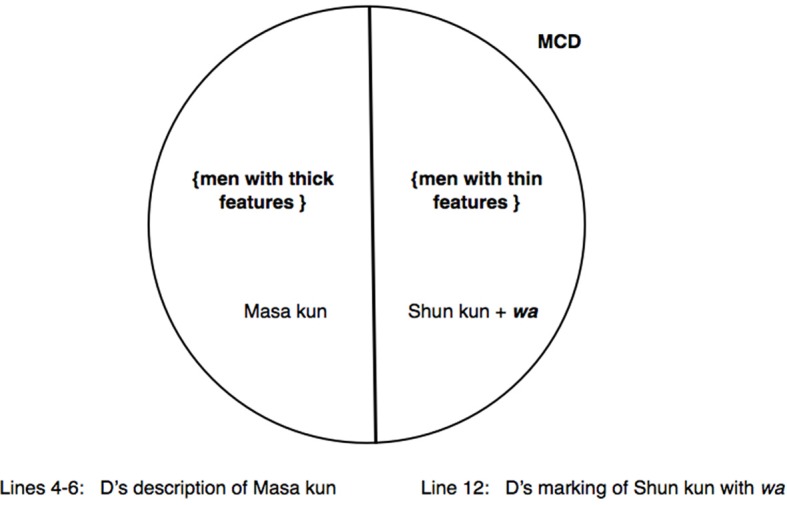
**Excerpt (7): The MCD jointly defined by participants by applying a binary opposition partitioning “eligible men” along the parameters “thick” or “thin” features**.

As with the previous excerpt, the preemptive action here is triggered and propelled by a constellation of factors. In addition to the unprojected opportunity for turn-entry created by a sound stretch on *wa*::, F's preemptive response is facilitated through extensive categorization activity prior to line 12, as detailed above. The explicit inclusion of Masa in the category {men with thick features} both prior to the beginning of the extract and in the extract itself establishes a firm foundation for categorizing additional members. What distinguishes this example from excerpts (2) and (6) is that while there was a potentially unspecified number of categories comprising the MCDs invoked in (2) and (6), the category {men with thick features} in the present example utilizes a binary opposition of thick vs. thin, thereby making relevant a MCD comprising two opposing categories and no others. Thus, the situated deployment of *wa* within an interactional environment in which the category {men with thick features} has previously been invoked serves as a ready mechanism for invoking the one and only possible co-class category—{men with thin features}. The resultant MCD is schematized in Figure 4.

As discussed previously, two different roles of *wa* have been identified by Maruyama (2003), namely, for performing opposite *or* parallel contrasts. It can be seen here that opposite contrast is the only type of contrast possible when a MCD is defined with reference to some binary opposition. Alternatively, some types of MCDs such as “types of medical doctors” inherently have many co-class categories, in which case, *wa* may trigger a selection from among the potentially multiple parallel categories rather than just one. In this sense, the examples inspected so far suggest that there is a higher order of generality that subsumes both roles under a single operation.

In extracts (6) and (7) above, a *wa*-marked reference formulation triggered an anticipatory completion or preemptive action that was quickly ratified by coparticipant(s). An inspection of the earlier talk revealed that crucial groundwork had already been laid through “adequate” preliminary categorization activities, including implicit or explicit invocation of some category and a candidate MCD. Such preparatory work was argued to underpin the formation of an interactional context ripe for further categorization work. It should come as no surprise, then, that a subsequent reference formulation marked with *wa* can create a fertile moment for triggering coparticipant anticipatory completion or preemptive action. The next section examines instances where a *wa*-triggered uptake is not ratified by the speaker, and explores how such developments may be linked to factors present in the preceding contextualization work.

## *Wa* used to mobilize a “search procedure” for a potential MCD when there is ambiguous or minimal contextual information

In the sequences examined above, there was little apparent contention among coparticipants with regard to the category and MCD being locally invoked, partly owing to the unequivocal contextualization work performed in prior talk. By way of contrast, the first excerpt to be scrutinized here exemplifies how a *wa*-marked reference formulation may make relevant multiple possibilities for MCDs due to ambiguities introduced in the immediate interactional environment. Nevertheless, a close tracking of the categorization work undertaken can reveal that participants display concord with respect to the kind of cognitive operation *wa* sets in motion. The final excerpt demonstrates that *wa* may trigger a preemptive action even when it is preceded by little or no preliminary categorization activity, suggesting that participants may resort to general cultural knowledge or “background expectancies” (Garfinkel, 1967) to furnish an independent basis for contextualization.

The following excerpt is from the same conversation as the one from which excerpt (6) was taken, which transpired when Mari and her daughter visited the home of a family friend Chie and her son Ken. Although too lengthy to show here, the categorization work within the excerpt can be understood against the backdrop of points raised earlier in the conversation, as outlined below in sequence:

- Ken has complained about having had little alternative but to be attentive to others' needs around the house and to be diligent with the housework (i.e., Ken ∈ {attentive people}).
- Ken has attributed his predicament to the fact that all the women around him (including Chie's close friend Kazuyo who often comes to stay at the house) are purportedly *suekko* “babies of the family,” further describing them as *noonoo to suru* “carefree” or “indolent” and completely reliant on Ken to serve them without themselves lifting a finger (i.e., Kazuyo ∈ {carefree people}).
- Mari has commented that men must nevertheless find such women utterly *kawaii* (i.e., lovable, sweet, cute, endearing, etc.).
- Chie then portrayed her friend Kazuyo as someone who has little self-awareness that everyone around Kazuyo may find her behavior bothersome (i.e., everyone around Kazuyo ∈ {people who find carefree behavior bothersome}).

(8) [YKH 1 34'23”-34'47”] Kazuyo's husband


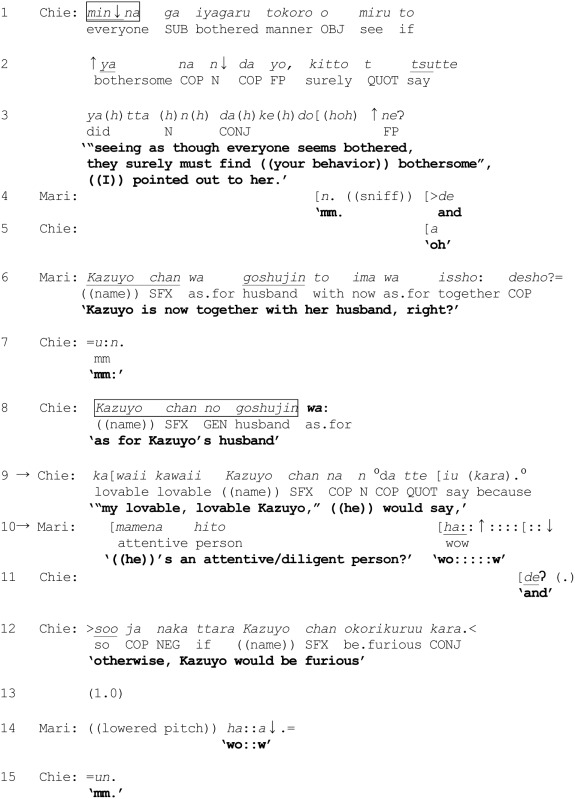


In lines 1–3, Chie uses direct reported speech to reenact her attempt to instill in Kazuyo an awareness that everyone must be bothered with her carefree behavior (point *d.* above), using an “extreme case formulation” (Pomerantz, 1986) *min'na* “everyone.” Mari then requests confirmation that Kazuyo is “now” with her husband (line 6) (apparently based on prior knowledge of Kazuyo's habit of leaving home, which is explicitly topicalized immediately following the present extract). This is quickly affirmed by Chie (line 7). Mari's move in line 6 can be heard in this specific context as a preliminary to enquiring if it is the husband who does all the housework, which would be contrary to conventional wisdom—an interpretation borne out by the way Mari subsequently performs an anticipatory completion in line 10, as discussed just below. However, before Mari has a chance to articulate the main question, Chie comes in with a new turn-beginning: *Kazuyo chan no goshujin wa:* “as for Kazuyo's husband,” by marking “husband” with *wa:* (line 8). As with excerpt (7), the sound stretch on *wa* not only serves as an unprojected opportunity for co-completion but also extends the duration of time for coparticipants to engage in the necessary cognitive operations locally precipitated by the *wa*-marked referent. Indeed, Chie and Mari almost simultaneously go on to complete Chie's turn-beginning. Interestingly, however, their respective turn-continuations are indicative of the invocation of divergent MCDs to partition the social world.

On the one hand, Chie completes her turn with an enactment of how Kazuyo's husband would hypothetically react: *kawaii kawaii Kazuyo chan na n* °*da tte iu (kara).*° “‘My lovable, lovable Kazuyo,’ ((he)) would say.” (line 9). Given that Chie has just claimed that everyone would be bothered (lines 1–3), to say the husband would find Kazuyo's behavior lovable is to treat the husband as an exception to this rule—i.e., that he would not find her behavior bothersome. In other words, Chie is building on the contextualization work performed by points *c.* and *d.* in choosing a MCD that partitions the population into two categories, by assigning the husband to the category {people who find carefree behavior lovable} in the co-class category of {people who find carefree behavior bothersome}.

**Figure 5:**
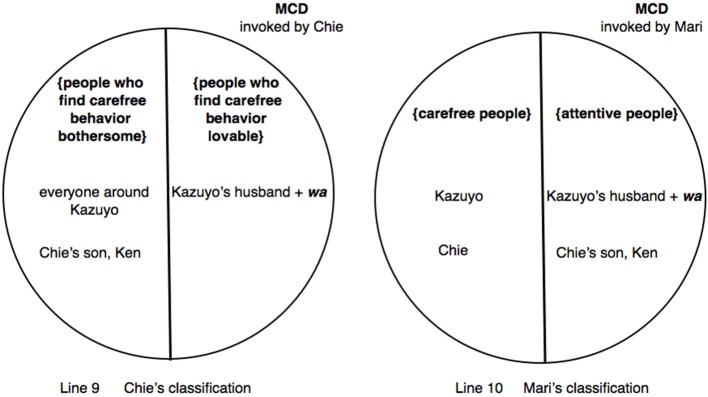
**Excerpt (8): Different MCDs triggered by Chie's marking of “Kazuyo's husband” with *wa.*** Chie invokes the MCD partitioned by *opposing reactions* to carefree behavior {people who find carefree behavior bothersome} vs. {people who find carefree behavior lovable} **(left)**, whereas Mari invokes the MCD defined by the binary opposition {carefree people} vs. {attentive people} **(right)**.

On the other hand, Mari's anticipatory completion “((he))'s an attentive/diligent person?” to characterize the husband (line 10) indicates that Mari has appropriated the slot made available by Chie's turn-beginning and pursued the main question projected by her own preliminary query in line 6, and has accordingly partitioned the same population differently. Mari puts the husband in the category {attentive people} which can be seen to be a co-class category of the previously invoked category {carefree people}, thereby orienting to a characterization of the husband which takes into account the prior contextualization work undertaken in points *a.* and *b.* Namely, Ken's earlier complaint about the women around him has made immanent the category {carefree people}, to which he has assigned Kazuyo, as well as the co-class category {attentive people} in which he has already included himself. Mari is now actively invoking these categories (which has until then only been immanent) triggered by Chie's deployment of *wa* (line 8). In sum, whereas Mari is dividing up the universe of discourse into a MCD consisting of opposing categories of attentive vs. carefree people, Chie can be observed to be orienting to the MCD defined by *reactions* to carefree behavior—consisting of opposing categories of {people who find carefree behavior bothersome} and {people who find carefree behavior lovable}. In other words, the concurrent completions by Chie and Mari in lines 9 and 10 respectively index and implement underlying cognitive operations that divide up the population in different ways. The categorization activities performed by Chie and Mari are schematized in Figure 5.

The above example illustrates how prior talk can sometimes make relevant multiple MCDs or ways of classifying the larger population. Indeed, if potential ambiguities are introduced by competing dimensions along which to categorize the population in prior contextualizing work, a situated *wa*-marked reference formulation may trigger disparate collaborative completions representing divergent projections of possible turn-trajectories. On a deeper level, however, the excerpt demonstrates that the apparent differences result from the implementation of the same basic cognitive operation mobilized by *wa* on empirically different MCDs. In this sense, excerpt (8) provides even greater warrant for the proposed operations of *wa*.

Alternatively, *wa* is sometimes occasioned to mark a referent in circumstances where there is minimal prior categorization activity to form a basis for identifying an overarching MCD being invoked. Excerpt (9) explores two further workings of *wa*. First, even where there is little preliminary categorization activity, the marking of a referent with *wa* may nonetheless serve as a trigger for coparticipants to make a “reasonable” guess of the categorization activity involved, by resorting to shared cultural knowledge or “background expectancies” (Garfinkel, 1967). Second, by building on such a prediction, participants can go beyond simply anticipating how a current speaker's turn might develop, and preemptively perform some relevant next action [as in excerpts (1) and (7)].

Japan is often described as a country where there is a persistent normative expectation to get married (to legally tie the knot) by a certain age, even though the average age at first marriage continues to rise (National Institute of Population and Social Security Research, 2011, Table 1-1, p. 2). The following excerpt from a reunion of members of a university yacht club (three women in their late twenties) presents a vivid commentary on the social and personal pressures that may drive one into marriage, even in spite of oneself. Aya, who is the only one out of the three who is already married, has just admitted to the others that her marriage was partly a result of an unremitting buildup of pressure making it difficult to go against the tide.

(9) [Mfriends 2685] Pressure to get married


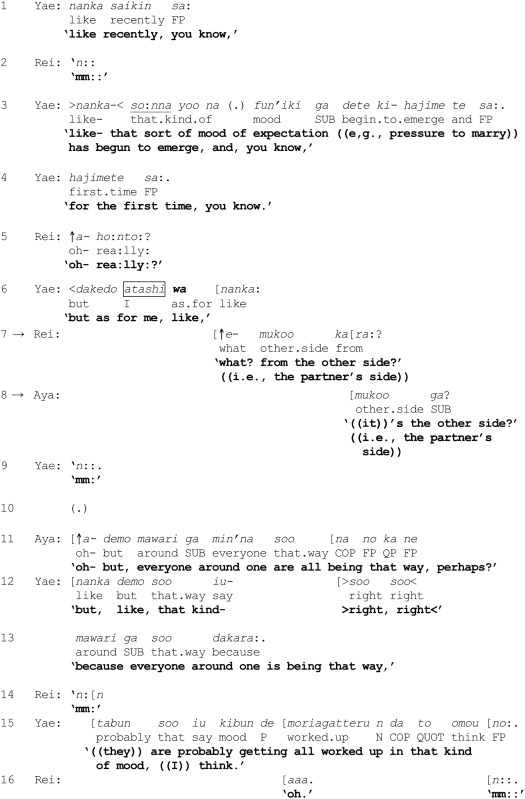


In lines 1 and 3, Yae begins a “second story” by reporting that the pressure for her to marry has likewise gained momentum: “like recently, you know, like- that sort of mood of expectation ((e.g., pressure to marry)) has begun to emerge, and, you know, for the first time, you know.” Rei treats this announcement as newsworthy in line 5 by employing a “change-of-state token” ↑*a*- “oh-” and pursues the informing: ↑*a*- *ho*:*nto*:? “oh- rea:lly?” (see Heritage, 1984). Yae then resumes her telling in line 6: *dakedo atashi wa nanka*: “but as for me, like,” using the contrastive conjunction *dakedo* “but,” which adumbrates a contrast, as well as marking *atashi* “I” with *wa*. Notably, this turn-beginning results in an immediate preemptive reaction from Rei: ↑*e*- *mukoo kara*:? “what? from the other side?” (line 7) containing ↑*e*- “what?” which, as noted previously, is regularly used to mark an informing as departing from one's expectation, supposition, prior knowledge or other orientation (Hayashi, 2009). In other words, without hearing how Yae's turn develops, Rei infers from Yae's marking of “I” with *wa* (line 6) that it is “the other side” (i.e., the partner's side) and not Yae herself who is the source of the pressure. Aya displays a similar understanding through her uptake in line 8: *mukoo ga*? “((it))'s the other side?” (i.e., the partner's side).

Drawing on the discussion so far on the role of *wa*, the marking of *atashi* “I” with *wa* (line 6) would be expected to trigger a search for a category from which “I” would be excluded, by retrospectively searching for some contextualization work in Yae's prior talk. In the excerpts examined previously, the marking of a referent with *wa* was preceded by prior categorization activity that participants could draw upon—such as the invoking of some category and a member of the category. In contrast, there is little if any prior categorization activity in the present excerpt, apart from the mention of the emergence of a mood of expectation that can potentially form the basis of defining a category such as {people creating mood of expectation}.

In the absence of adequate contextual information, the coparticipants appear to base their subsequent categorization activities on background expectancies. The fact that Rei and Aya both identify *mukoo* “the other side” (i.e., the partner's side) as the source of the pressure suggests that the search procedure may have proceeded roughly along the following lines:

- On reexamining Yae's prior talk, the coparticipants locate the category {people creating mood of expectation}, though Yae has not specified any member of the category.
- The appearance of *dakedo* “but” and the marking of “I” with *wa* (line 6) can be used to exclude Yae from the category {people creating mood of expectation}, thereby implying that Yae ∈ {people not creating mood of expectation}.
- The binary opposition in step *b.* leads to a search for specific person(s) who may be the source of the mood in an overarching MCD.
- Based on background expectancies and conventional wisdom that there are only two parties to a marriage (i.e., Yae and her partner), the coparticipants select the MCD “parties to a marriage” consisting of two categories {ego's side} and {partner's side} which is “duplicatively organized,” i.e., that the set of categories define a social unit (Sacks, 1986, p. 334).
- The coparticipants appropriate the MCD identified in step *d.* above, and superimpose the structure of this MCD (a binary opposition) over the MCD identified in steps *a. – c.* above in order to discover the source of the mood of expectation. As it has already been established (in step *b.* above) that Yae∈{people not creating mood of expectation}, the coparticipants arrive at the conclusion that Yae's partner is the source of the mood—i.e., assigns the partner to the category {people creating mood of expectation} (lines 7 and 8).
- Consequently, all responsibility for exerting the pressure to marry is attributed to Yae's partner.

It appears that the coparticipants have not only anticipated the trajectory of Yae's turn-beginning in line 6, but have implicitly built on it to initiate their preemptive reactions in lines 7–8.

There is, nevertheless, little guarantee that a “search” will necessarily be endorsed by the original speaker, and “(o)f course, using that procedure for finding the category, you may never come across occasions for seeing that it's ‘incorrect”’ (Sacks, 1992, Vol. I, p. 337). However, in excerpt (9) an occasion to (in)validate the coparticipants' choice of MCD is afforded. But first, it should be noted that the reactions of Rei and Aya in lines 7–8 contain a potentially problematic inference that the partner may be pressuring Yae to get married against her will. Perhaps in order to counter such an inference, Yae simply proffers a minimal acknowledgement (line 9) followed by a micro-pause (line 10), hearable as implicating some interactional trouble. Indeed, just as Yae begins in line 12 to produce a potential disagreement using the connective *demo* “but” (Mori, 1999), Aya simultaneously comes in (line 11) to treat the minimal response as pointing to a problem with the presumptive inferences drawn earlier by Rei and herself in lines 7 and 8 respectively. In other words, Aya locates the problem as one involving a failed search for an appropriate MCD in the previous turns, i.e., the invocation of the device, “parties to a marriage.” This is partly evidenced by Aya's modified formulation in line 11, which is a renewed attempt at searching for another, more “suitable” MCD: she begins with a change-of-state token ↑*a*- “oh-” followed by the activation of an alternative MCD, “everyone around one.” Whereas the previous MCD “parties to a marriage” was sharply defined through a binary opposition, the new MCD is diffuse and blurs the earlier distinction between the two parties to marriage—for instance, whether it includes just the couple, their immediate family members, a still wider circle of relatives, friends and acquaintances of the families, or for that matter, even shading into the amorphous notion of *seken* “society at large.”

**Figure 6:**
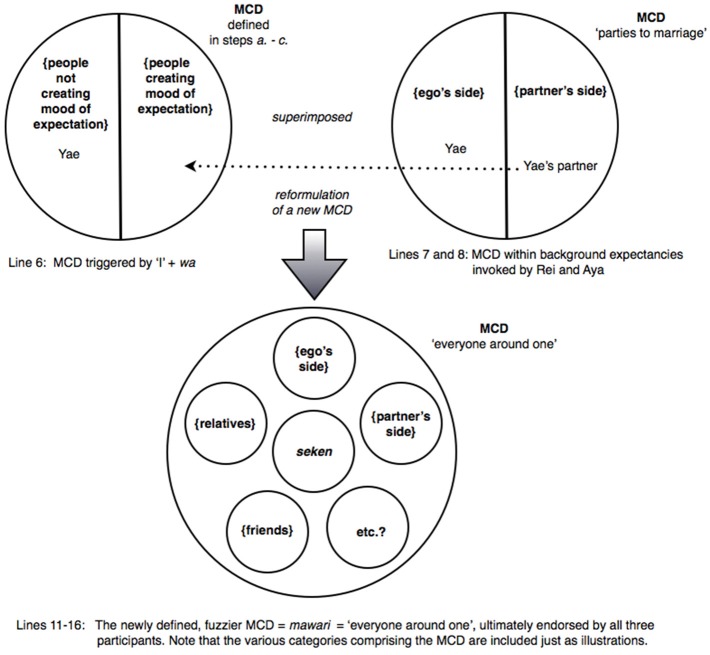
**Excerpt (9): Yae's marking of “I” with *wa* triggers a MCD consisting of categories {people not creating mood of expectation} and {people creating mood of expectation}, with Yae belonging to the former (top left)**. Rei and Aya superimpose a MCD retrieved from their background expectancies in order to discover the source of the mood of expectation to marry **(top right)**. However, Aya and Yae reformulate the just proposed MCD into a fuzzier MCD, thereby diffusing the source of the mood (bottom).

One consequence of invoking this new device is to drain away some of the responsibility for creating the mood of expectation from the partner, and to redistribute it among a broader and fuzzier collection of people. The revised MCD (and the resultant redistribution of responsibility) is now ratified enthusiastically by Yae herself: >*soo soo*<*mawari ga soo dakara*:. *tabun soo iu kibun de moriagatteru n da to omou no*: “>right, right < because everyone around one is being that way, ((they)) are probably getting all worked up in that kind of mood, ((I)) think.” (lines 12–13 and 15). Interestingly, Yae's talk diffuses the source and nature of the mood of expectation even further, and is rendered highly tentative through expressions such as *omou no* “((I)) think,” *tabun* “probably,” and the use of anaphoric expressions *soo dakara* “being that way” and *soo iu kibun* “that kind of mood,” thereby articulating a sense of ambivalence about the elusive yet pervasive societal pressure to get married. Rei also shows recognition and acceptance of the reformulated MCD in line 16. All told, the newly defined, diffuse MCD “everyone around one” jointly reformulated by Aya and Yae is ultimately endorsed by all three participants. The series of categorization activities performed in this extract is schematized in Figure 6.

To summarize, Excerpt (9) exemplifies the deployment of *wa* in an interactional environment preceded by minimal categorization work. Even in such instances, the marking of a referent with *wa* may set in motion a search procedure for a category containing the referent and an overarching MCD. Where there is little prior categorization work to serve as a basis for the search, participants may consult their cultural knowledge and background expectancies as a basis for implementing the cognitive operations of *wa*.

## Concluding comments

This article investigated the potential of *wa* to propose, trigger, and propel anticipatory completions and preemptive action trajectories within locally emergent frames of interaction incorporating interlinked membership categorization activities. The mobilization of *wa* is often preceded by earlier classification activities such as assigning a member of a population to some category. Marking a referent with *tte* represents a typical method to explicitly invoke a new category while simultaneously proposing membership of the referent in that category. More generally, an explicit or implicit invocation of a category and a member of the category through *some* means can create an interactional environment that makes salient extended opportunities for subsequent interlocking categorization activities, which are regularly performed through the differential use of *mo* and/or *wa*. Specifically, while *mo* is used to include another referent in a category that has already been invoked, the data indicate that marking a referent with *wa* indexes a cognitive operation to exclude the referent from an already invoked category and to assign it instead to a contrastive co-class category in a relevant membership categorization device. Detailed examination of instances where the situated marking of a referent with *wa* leads to anticipatory completions and preemptive actions yielded evidence that participants draw on such underlying categorization operations to project the trajectory of the turn-in-progress and to plan a relevant next action.

The projective potential of *wa* has been explored in a range of interactional contexts. First, when progressive groundwork is laid through preliminary contextualization work, participants can develop an increasingly firm basis on which to mobilize the capacity of *wa* to pick out a co-class category from a relevant MCD, and achieve consensus as to how to classify a *wa*-marked referent. Further, the proffering of a *wa*-marked referent is routinely accompanied by a hitch in progressivity through a sound stretch on *wa* or through the use of fillers such as *nanka*, extending the duration of time available for cognitive processing as well as providing “unprojected opportunities” for entry into the turn-space of the current speaker (see Lerner, 1996). I have argued that the categorization operations implemented by *wa*, together with preparatory contextualization work and temporal-productional features may reach critical mass, and trigger coparticipant anticipatory completions and preemptive actions.

On the other hand, where potential ambiguities are introduced through the immanence of multiple MCDs in the immediate interactional environment, a *wa*-marked referent may engender the relevance of disparate MCDs, representing divergent ways of partitioning members of a population. Nevertheless, inspection of the categorization operations coparticipants perform through *wa* can paradoxically indicate that they are implementing an identical cognitive operation, albeit on different MCDs. Such instances can serve as “deviant case analysis” to further warrant the proposed role of *wa*. Finally, even in cases where no category or MCD has been explicitly invoked in prior talk, the marking of a referent with *wa* may sometimes set in motion a search procedure for a possible MCD containing a category from which the referent is excluded. When there is only minimal contextualization work to draw on within the immediate interactional environment, participants may resort to cultural or background knowledge such as relevant “standardized relational pairs” in order to presumptively identify a likely MCD. The basic categorization operations identified in this study are outlined as algorithms in Table 1.

**Table 1:** **Algorithms**.

Algorithm 1	If *y* is a member of a population and *Y* is a category (or a description of a category), then “y *tte Y*” can invoke the category *Y*, and propose the incumbency of *y* in *Y* (denoted *y*∈*Y*).
Algorithm 2	For a member of a population *y* and a category *Y*, if it has been established that *y*∈*Y*, then the subsequent marking of another member of the population *x* with *mo* “x *mo*” can assign *x* to the same category *Y* (i.e., *x*∈*Y*).
Algorithm 3	For a member of a population *y* and a category *Y*, if it has been established that *y*∈*Y*, then the subsequent marking of another member of the population *x* with *wa* “x *wa*” can exclude *x* from the category *Y* and simultaneously propose the existence of another category *X* to which *x* belongs, *and* a membership categorization device *M* in which *X* and *Y* are co-class categories (i.e., *X* is in the complement of *Y* in *M*).
Corollary to Algorithm 3	As a special case of Algorithm 3 above, if a category *Y* has been defined in such a way as to set up a binary opposition, then the membership categorization device *M* proposed will consist of only two categories *Y* and *X*, where *X* = ~*Y* (i.e., *X* is equal to the complement of *Y*)
Algorithm 4	For a member of a population *x*, if *wa* has been used to mark *x* but no membership categorization device has been implicitly or explicitly specified, then “*x wa*” may activate a “search procedure” to identify a membership categorization device *M* containing categories *X* and *Y* such that *x* ∈ *X* and *Y* is a co-class category of *X* in *M*.

The picture of *wa* which emerges here is as a resource deployed to assemble together a myriad of features in the moment-by-moment unfolding interactional environment toward activating and projecting a specific type of categorization activity, which can compensate for the tendency toward delayed projectability in Japanese conversation (see Tanaka, 1999, 2000). If one were to grant that this portrayal can serve as a realistic model of the actual workings of *wa*, it should be apparent that an enquiry that limits consideration to written or non-interactional data would be unable to capture the extent of the complex processes it points to. The operations enabled by *wa*, which have been a subject of an agelong debate in linguistics, appear to exhibit a remarkable order of systematicity when investigated *in situ* through the lens of conversation analysis and membership categorization/set theory. In this regard, anticipatory completions and preemptive actions offer an indispensable vehicle to catch such processes “in flight,” as they provide coparticipants' online commentary on the cognitive processing through which an upcoming trajectory of a turn is being projected and acted upon in the middle of the turn. Particularly revelatory are collaborative completions where two participants concurrently display how they are processing and analyzing one and the same *wa*-marked turn-beginning [such as excerpt (8)]. The fact that the completions occur simultaneously is proof that their respective projections were arrived at independently.

The capacity of “topic particles” has often been cited as a characteristic and prominent feature of the Japanese language to grammatically distinguish a “topic” of discourse from the grammatical subject (see Kuno, 1973; Maynard, 1981, 1987; Hinds et al., 1987; Shibatani, 1990; Noda, 1996; Iwasaki, 2013b), along with some other Asian languages such as Korean and Singaporean English (see Deterding, 2007, p. 61; Leimgruber, 2011). According to Sidnell and Enfield (2012), “some social actions are more readily carried out, or are carried out in specific ways, by speakers of a given language by virtue of the lexicogrammatical properties specific to that languages” (p. 312). As a consequence, the language-specific lexicogrammatical resources used to accomplish particular actions can introduce “collateral effects and in this way give the action a local spin or inflection” (Sidnell and Enfield, 2012, p. 302). The apparently dynamic role of *wa* (and other “topic particles”) to project turn-trajectories by implementing categorization activities invites further investigation as a possible “collateral effect” of the lexicogrammatical resources made available in Japanese. Though beyond the purview of this article, a preliminary inspection of the data suggest that participants routinely utilize topic particles for various other, related classifying activities, including negotiating modifications to the definition of a proposed category, adding or deleting members from a category, and mobilizing a search procedure for alternative categories and MCDs, etc. Future cross-linguistic studies on interactional resources used to render visible and analyzable the contingent categorization work oriented to by participants may hopefully serve as stimuli in the exploration of hitherto untrodden terrains of membership categorization activities through comparison of tools available in different languages for engaging in the most human and universal of social actions, namely jointly categorizing the world around us (e.g., Lévi-Strauss, 1969).

### Conflict of interest statement

The author declares that the research was conducted in the absence of any commercial or financial relationships that could be construed as a potential conflict of interest.

## Footnotes

^1^F accomplishes this in part by leaving unexpressed any first person marking and partly through use of the copula *da* to mark the assessment as her own. Szczepek (2000a,b) discusses a similar practice in English of “borrowing” the construction of a prior turn to produce one's turn.
