# Supplementary material for: Action-projection in Japanese conversation: topic particles wa, mo, and tte for triggering categorization activities
Source: Front Psychol. 2015 Aug 26;6:1113. doi: 10.3389/fpsyg.2015.01113 (PMC4549552; doi:10.3389/fpsyg.2015.01113)
Supplement: Supplementary file 1 [file DataSheet1.DOCX]

**Appendix**: Transcription conventions

The original Japanese talk appears in line 1 of the transcripts, word-by-word translation/grammatical description in line 2, and an English gloss in line 3. Double parentheses are used not only to encase transcriptionists’ comments but also for supplying unexpressed elements in the English gloss in line 3. A period between words in the word-by-word translation in the interlinear gloss indicates that the English translation of a Japanese word consists of two or more words. The referent marked with a topic particle is encased in a border, and topic particles under scrutiny are highlighted in boldface.

*Abbreviations in transcripts*

AP appositional

COMP complementizer

CONJ conjunctive particle

COP copula

DF dysfluency

FP final particle

GEN genitive particle

INS instrumental particle

LOC locative particle

N nominalizer

NEG negative

OBJ object particle (accusative)

P particle

PASS passive

QP question particle

QUOT quotative particle

SFX suffix

SUB subject particle (nominative)

TOP topic particle

Other symbols used

ʔ glottal stop

{ } category or set

[ ] membership categorization device or collection of categories

a ∈ A a is an element of the category/set A

Transcription of visual conduct in excerpt (6):

|transcribed talk ‘|’ denotes the location where visual conduct

|((description of visual conduct)) starts in relation to the transcribed talk
